# Non-Puerperal Uterine Inversion

**DOI:** 10.5334/jbr-btr.974

**Published:** 2016-03-10

**Authors:** Isabelle Leconte, Cecile Thierry, Antony Bongiorno, Mathieu Luyckx, Latifa Fellah

**Affiliations:** 1 1Cliniques Universitaires Saint Luc, BE

**Keywords:** uterus, inversion, MRI

## Abstract

We report a case of non-puerperal uterine inversion, illustrating the correlation between MRI and pre-operation macroscopic images.

Non-puerperal uterine inversion is a rare condition that can be easily diagnosed with MRI. This article reports a case of non-puerperal uterine inversion, illustrating the correlation between MRI and intra-operative images.

A 56-year-old postmenopausal woman presented with increased, painless bleeding for 10 days. Laboratory tests revealed a hemoglobin level of 9g/dL. The patient had not been seen by a gynecologist for 6 years. Pelvic examination revealed the presence of a black, brown, and white voluminous vaginal mass of approximately 8 cm in diameter along with a small amount of red-brown vaginal bleeding. A transvaginal ultrasound was difficult due to the presence of the mass in the vagina, but the mass was thought to be continuous with the cervix. The uterus and ovaries were not identified separately.

A malignant mass was suspected, and an MRI was requested. It confirmed the presence of an intravaginal mass measuring 10 cm in diameter. The T2-weighted coronal images showed the uterine corpus in a U-shape above the mass (Figure 1a). The cervix surrounded the uterine corpus, and the vaginal fornix surrounded both the corpus and the cervix. The T2-weighted axial images therefore disclosed, from the center outwards, the uterine corpus, the cervix, and the fornix (target sign) and the adnexes and round ligaments bulging centrally out the top of the uterus in a bullseye appearance (Figure 1b). The T2-weighted sagittal images showed one ovary directly above the cervix (Figure 1c). On T1-weighted images, a few high-signal intensity spots were detected within the mass, persisting upon fat saturation, corresponding to hemorrhages (Figure 2a). The signal was heterogeneous after the injection of gadolinium (Figure 2b).

**Figure 1:**
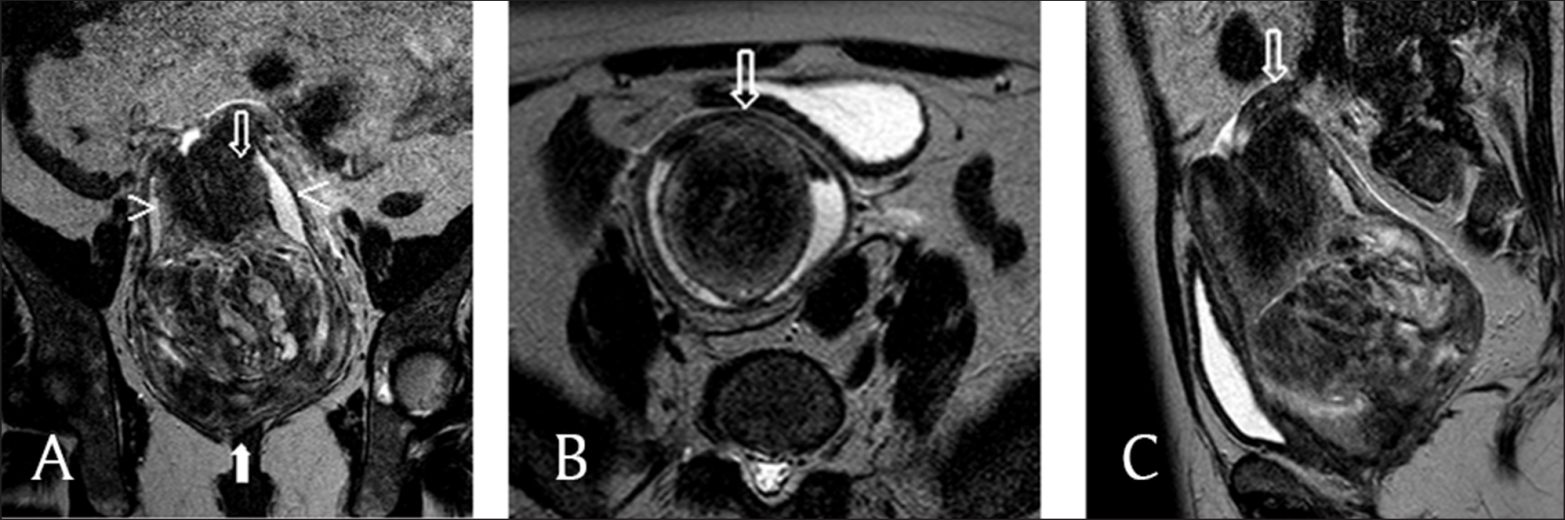
T2-weighted MRI (a, b, c) **a.** This coronal image shows a vaginal heterogeneous mass (filled arrow), with the uterine corpus in a U-shape above the mass (empty arrow). The cervix surrounds the corpus, and the vaginal fornix surrounds both the corpus and the cervix (arrowheads). **b.** This axial image shows, from the center outwards, the uterine corpus, the cervix, and the fornix and the invaginated round ligaments in a bullseye appearance (arrow). **c.** This sagittal image shows one ovary above the cervix (arrow).

**Figure 2:**
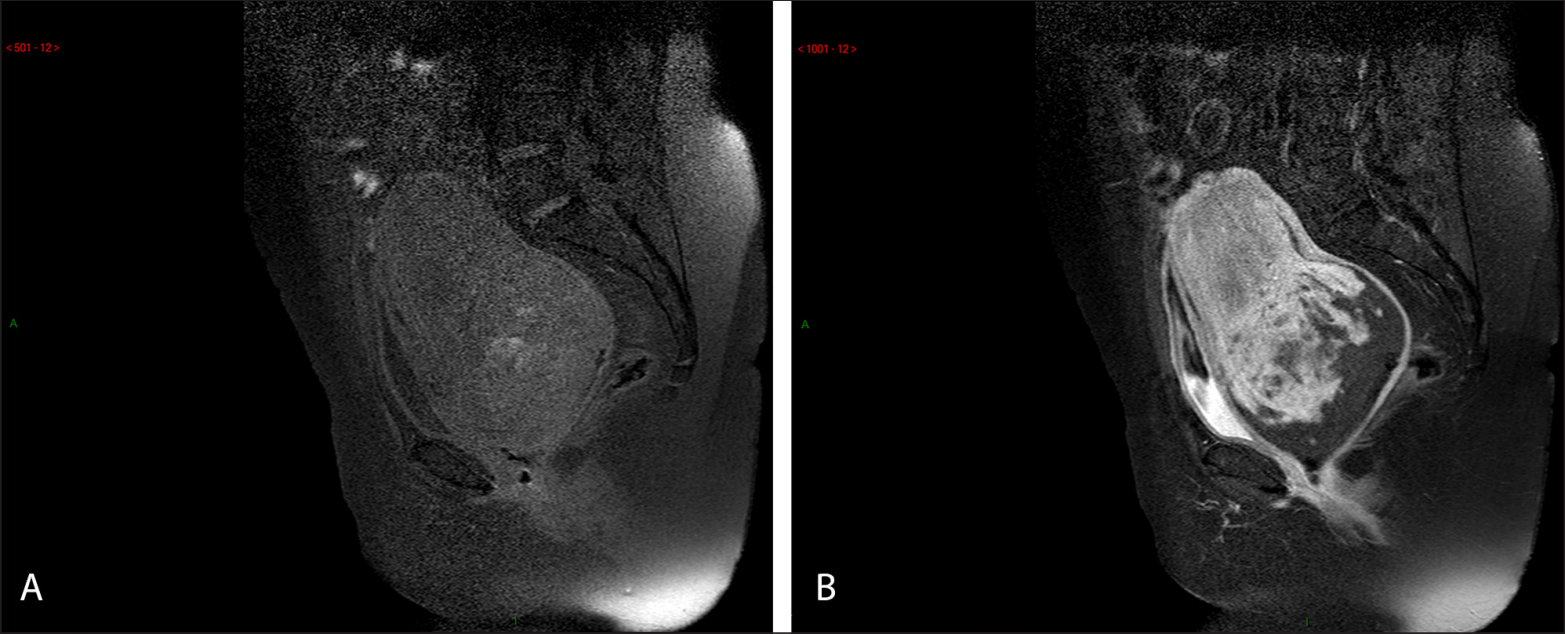
Sagittal T1-weighted (a, b) **a.** The bright spots of suppressed fat in the image denote hemorrhagic areas. **b.** After an injection of gadolinium, there is a heterogeneous enhancement of the mass.

A hysterectomy was performed. Histopathology revealed a leiomyoma in the fundus in an advanced state of necrosis, leading to uterine inversion, that is, the uterus is turned inside out. The voluminous mass had descended through the endocervical lumen and cervix, pulling with it the uterine corpus, which reversed in the vagina, followed by the fallopian tubes, the round ligaments, and the ovaries (Figure 3a).

**Figure 3:**
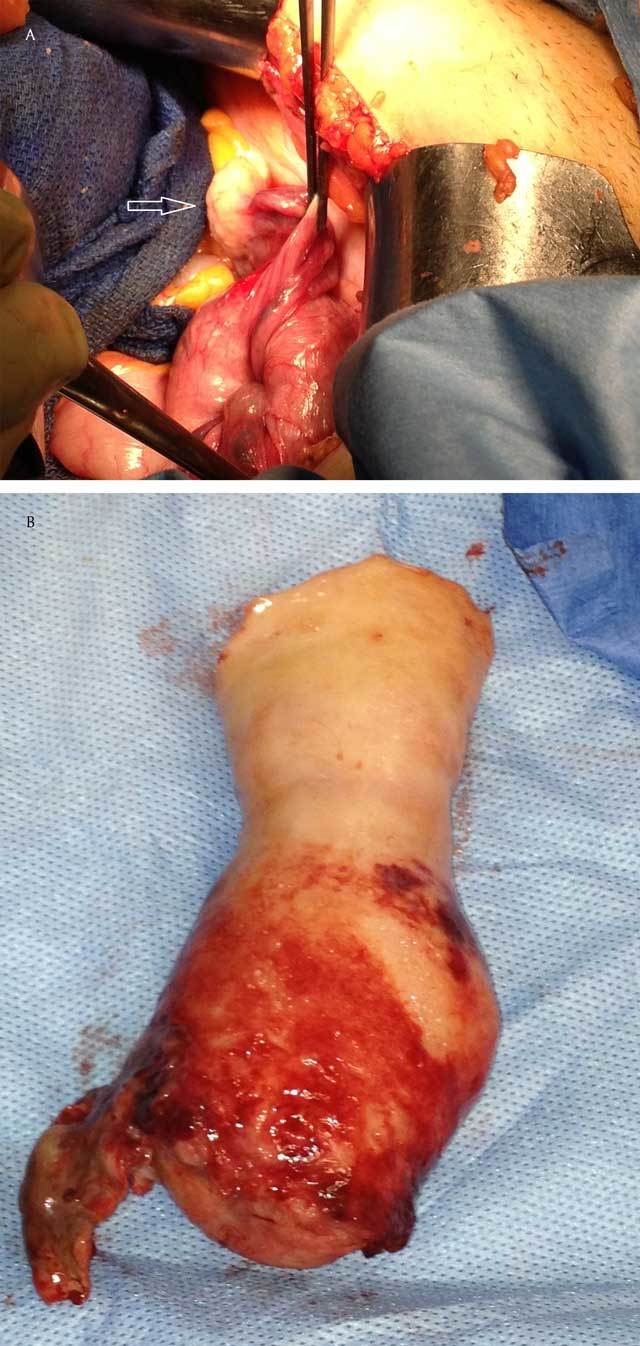
This operative view during a laparotomy shows the fallopian tube and one of the ovaries (arrow) outside the inverted uterus (a) and a completely reversed uterus after resection (b).

## Discussion

Uterine inversion is a rare condition. The incidence of puerperal uterine inversion is estimated to be 1/30,000 deliveries and is considered a serious complication of child delivery [1]. Non-puerperal uterine inversion is a rarer condition occurring in approximately 17 percent of all uterine inversions. One hundred and fifty cases of non-puerperal uterine inversions were documented from 1887 to 2006 by Gomez-Lobo et al. [2].

Non-puerperal uterine inversion may be idiopathic or associated with such predisposing factors as benign uterine tumors in 70–80 percent of cases (leiomyoma, endometrial polyps), or malignant tumor in the remaining, especially in young women (leiomyosarcoma, mixed Müllerian sarcoma, rhabdomyosarcoma, endometrial and cervical carcinoma) [1,2,3,4,5,6,7,8,9,10,11,12,13,14,15,16,17]. The degree of inversion may be classified into incomplete, complete, or total. In the incomplete form, the uterine fundus descends inferiorly but not through the cervix. In complete inversion, the fundus and corpus extend through the cervix. In total inversion, the vagina is also inverted [16].

The mechanism of uterine inversion has not been clearly identified. The most common factor seems to be a large submucosal mass in the fundus. The mass is generally attached to the uterus via a thin pedicle. The walls of the uterus may become thin and weak due to the presence of the mass. Thinned walls may be pulled down by the weight of the mass, while uterine contractions may also play a role in this mechanism. The distension of the uterine cavity may lead to a dilation of the cervix, causing the expulsion of the mass. It has been suggested that additional processes that raise intra-abdominal pressure including coughing, sneezing, and straining may be involved in the development of uterine inversion [17].

The diagnosis is not easy if based on physical examination alone. The acute form is defined by strong symptomatic evidence, while the chronic form can be asymptomatic or associated to pelvic pain with a sensation of heaviness or bleeding [3]. Anemia, urinary dysfunction, and vaginal mass are also described. Hemorrhage is unusual, unlike cases of puerperal uterine inversion. Upon physical examination, a vaginal mass can be detected, but the uterine fundus is not palpable by bimanual examination. The differential diagnosis is prolapsing uterine or cervical mass.

Transvaginal ultrasound shows only the mass. Transabdominal ultrasound usually shows a mass at the cervico-vaginal level, and the uterine morphology is difficult to define due to the mass. However, two signs are described: the indentation of the fundal area and the depressed longitudinal groove extending from the fundus to the center of the inverted portion [18,19].

CT is of limited value due to its relatively poor soft tissue contrast. However, it can be used when MRI is not possible, with a contrast agent to delineate the mass and uterus.

If suspected, MRI is the best imaging modality to diagnose uterine inversion, showing, as in the presented case, a U-shaped uterine cavity on the sagittal and coronal images and a reversed uterine fundus (target sign) and a bullseye aspect on the axial images [5,20]. Moulding and Hawnaur suggest that the key to identify uterine inversion on MRI is to recognize the round ligaments and fallopian tubes bulging centrally out the top of the uterus [21]. Indeed, these structures are pulled downwards and medially by the inverting fundus. Furthermore, MRI can characterize the mass involved and depict the changes of the uterus.

A conservative treatment may be approached if the inversion can be reduced, that is, in cases of incomplete inversion [3]. Similarly, in young women with a suspected benign tumor, a conservative treatment will be pursued until the histopathology confirms the benign nature of the mass. In all other cases, treatment will generally consist in hysterectomy.

## Competing Interests

The authors declare that they have no competing interests.
